# A Small Device May Deliver King-Sized Solutions for Patients With an Exacerbation of Cystic Fibrosis

**DOI:** 10.1155/ijpe/9184954

**Published:** 2024-11-21

**Authors:** P. Laird, G. MacKenzie, F. Gill, C. Burr, E. McKinnon, M. Cooper, E. Geelhoed, A. Schultz

**Affiliations:** ^1^Physiotherapy Department, Perth Children's Hospital, Perth, Western Australia, Australia; ^2^Walyan Respiratory Research Centre, Telethon Kids Institute, University of WA, Perth, Western Australia, Australia; ^3^The School of Nursing, Curtin University, Perth, Western Australia, Australia; ^4^Department of Respiratory and Sleep Medicine, Perth Children's Hospital, Perth, Western Australia, Australia; ^5^School of Allied Health, University of Western Australia, Perth, Western Australia, Australia

**Keywords:** cystic fibrosis, family-centred care, spring-infusor

## Abstract

**Aim:** The aim is to examine whether using a portable spring-infusor device to deliver antibiotics compared with a standard infusion pump (SIP) translated to (i) improve health outcomes, (ii) reduce the length of stay (LoS), and (iii) reduce cost for treatment of exacerbations of cystic fibrosis (CF).

**Methods:** An observational cohort study was conducted between December 2020 and June 2022 with participants aged 8–19 years admitted for exacerbation of CF. An activity monitor was fitted to participants to measure physical activities for the final 5 days of hospital admission. LoS was measured (days). Group allocation was according to participant preference. Costs were compared between the two groups for LoS, pump cost, and preparation and administration of antibiotics.

**Results:**Twenty-seven of 30 eligible participants were approached, and 22 consented. Data were captured for 16 participants (spring-infusor *n* = 9): 10 female; mean (SD) age 14.5 (2.1) years. Average step count was negatively associated with age (rho = 0.50), and greater overall in participants using spring-infusors (mean 5324 (SD 2873) steps) versus SIPs (4806 (3116) steps) - mean (95% CI) increase in the spring-infusor group of 3246 (54-6438) for participants of the same age. LoS was longer on average in the SIP group, (mean (SD) LoS: 16.1 (4.3) versus 12.4 (1.7)). The estimated cost saving for a child using a spring-infusor was AUS$12,000.

**Conclusion:**Results from the study suggest that children hospitalised for exacerbations of CF are more active if they receive antibiotics via a spring-infusor device compared with a SIP, and have reduced hospital stay that results in cost saving to the hospital. *What is already known?* Spring-infusors are small, portable, and mechanical devices to deliver intravenous antibiotics to patients. Spring-infusors are preferred by patients with CF at Perth Children's Hospital Physical activity in children with CF is recommended, including during hospital admissions to promote wellbeing, quality of life, and health outcomes. *What this paper adds?* Children hospitalised for exacerbations of CF may be more active if they receive antibiotics via a portable spring-infusor device compared with a SIP. Children using spring-infusors had reduced hospital stays that results in cost saving to the hospital. Children hospitalised for exacerbations of CF step on average, fewer than 5000 steps per day, which is well below recommendations.

## 1. Background

Historically, cystic fibrosis (CF) has been characterised by a severe and life-limiting genetic disease [1]. However, today, people with CF are living longer [2]. Improvements in life expectancy are due to multiple advances in therapeutic management, which impose a large treatment burden on patients and their families [1]. The treatment burden includes complex and time-consuming regimes of daily medications, airway clearance, and exercise. The complexity of managing CF can negatively impact emotional wellbeing, quality of life, adherence to treatment, and ability to maintain usual activities. Respiratory exacerbations (acute deterioration of respiratory status, typically characterised by a worsening of cough, drop in lung function, and other signs and symptoms [3]) can increase the burden of living with CF and can result in hospitalisation. Patients are usually hospitalised for 10–21 days and receive intravenous antibiotic therapy (IVAB) [1]. During an admission, multiple IVAB infusions can be required each day. The total daily time for infusions can exceed 4 h, which can impact treatment burden, mental wellbeing, and activity levels [4].

Reduced activity levels and exercise tolerance are some of the systemic consequences of CF. Further, hospitalisation for respiratory exacerbations is associated with less physical activity [5]. In contrast, higher activity levels have been shown to be associated with improved participation in activities of daily living and quality of life and reduced hospital admissions for patients with CF [6, 7]. Therefore, reducing barriers to ease of mobility for patients admitted to the hospital for exacerbations of CF may result in increased activity levels and reduced length of stay (LoS).

The IVAB administration device may significantly impact on the mobility levels of patients, particularly if infusions exceed 4 h a day. It is feasible that a patient connected to a standard electronic infusion pump may be less mobile than someone connected to a portable device. Spring-infusor (Go Medical Industries, Pty Ltd, Perth, Australia) devices are portable devices and show promise in promoting greater mobility and improving patient wellbeing [4]. Spring-infusors are small, mechanical spring-driven syringe pumps and are lightweight, allowing patients to maintain mobility during IVAB therapy. Spring-infusors are effective and safe [8]. Spring-infusors were previously widely used by patients with CF over a two-decade period at Perth Children's Hospital, the only tertiary hospital for children and young people in Western Australia. However, in recent years, the use of the device declined and became ad hoc. We previously investigated why the use of the device declined and if its reintroduction was feasible [4]. The reasons for the reduction of its use included some misconceptions or rumours. Firstly, there was a belief that the device was not safe and secondly that the device contributed to peripheral line failures. Both perceptions were not substantiated by factual evidence [4]. In contrast, we found that patients preferred spring-infusors due to improved mental wellbeing (via a less visible reminder of “illness,” greater autonomy, and no alarm) and improved mobility [4]. Further, many nurses reported that patients preferred the device, with patient and family-centred care being a priority for nursing staff [9]. Given the preference for using the device and identifying its safety (approved for use by the Therapeutic Goods Administration), we sought to investigate for potential benefits.

Therefore, our aims were to compare spring-infusor devices and standard infusion pumps (SIPs) for routine IVAB administration during hospital admissions for respiratory exacerbations of CF in children and young people. Specifically, we aimed to investigate if there was any association with spring-infusor use and

1. Health outcomes, including increased activity levels (via step count), quality of life, and sleep time.
2. LoS
3. Health economic cost

We hypothesised that patients using spring-infusors would have

1. Improved health outcomes as measured by higher step counts, improved quality of life scores, and improved sleep time,
2. Shorter LoS, and
3. Reduction in health economic cost compared to patients using SIPs.

## 2. Methods

An observational cohort study was conducted between December 2020 and June 2022 at Perth Children's Hospital.

### 2.1. Study Setting

The Perth Children's Hospital in Perth, Western Australia, is a 298-bed public hospital catering for children and young people and is the only specialist CF centre in the state, providing multidisciplinary care for approximately 200 children with CF.

### 2.2. Recruitment

Patients were eligible if they had CF, were aged 8–19 years, and were admitted to the hospital for a respiratory exacerbation. Potential participants with CF or their parent/guardian were approached in person by a clinician–researcher at the beginning of their hospital admission. Written informed consent was obtained for all patients and their parents/guardians for those patients under 16 years of age. The study was approved by the Child and Adolescent Health Service Ethics Board (RGS 3880).

### 2.3. Procedure

Once recruited, the participant's mode of delivery was documented, that is, a spring-infusor or SIP. Generally, adolescent patients admitted to the adolescent ward were given a choice of device. Those patients admitted outside of the adolescent ward were not routinely offered spring-infusors, and therefore, the use of the device on nonadolescent wards was more ad hoc. However, those patients were able to request to use a spring-infusor. Spring-infusor use was subject to availability.

A different clinician–researcher then fitted the participants with the activity monitor and provided the questionnaires and activity diary. The activity monitor (ActivPAL4 PAL Technologies Ltd, Glasgow, UK) was fitted to the midthigh of patients using an adhesive tape and waterproof dressing to allow showering. The ActivPAL4 is a small device (35 × 55 × 7 mm, 20 g) and is a valid and reliable assessment tool for distinguishing and measuring postures and step counts in children [10]. The ActivPAL4 can distinguish between positions of standing (vertical position) and sedentary (sitting or lying (horizontal position)) and transitions from sit to stand due to the location on the participants' thigh [11]. The device uses an accelerometer and contains a microprocessor, sensing element, and recording element. The patient wore the monitor for at least the final five consecutive days of their admission [12]. The chip was then collected from the patient, and data was downloaded into the PALconnect software programme. Activity data were retrieved for the final 5 days of the patient's admission and entered into REDCap [13]. The ACTIPAL4 does not accurately distinguish sleep time from lying time or the number of awakenings [14]. Therefore, patients filled out a daily activity diary (Appendix A) to correlate ACTIPAL4 data with patient reports for time sleeping and account for any activity undertaken if the device was removed. A day of activity included all activity recorded outside of sleep time. Lung function data were collected on all participants. Forced expiratory volume in 1 s as a percent of predicted (FEV_1_pp) was collected on admission and discharge, with the percentage change in lung function calculated, that is, FEV_1_pp change from the beginning and end of the admission.

These clinician–researchers were not blinded to the drug delivery mode as the participant may have been receiving IVABs at the time the researchers were either recruiting or fitting the device. However, the data were analysed by a statistician who was not involved in any recruitment, data collection, or data entry.

Patients filled out the CF questionnaire-revised (CFQ-R) on admission and discharge. The CFQ-R is a disease-specific and validated tool to assess health-related quality of life in patients with CF [15]. The CFQ-R is divided into several domains, depending on the child's age. The domains analysed for this study were treatment burden, physical status, respiratory status, and emotional status, which were available in all age versions of the CFQ-R. The scores for each of the domains are out of 100% with higher scores representing an improvement in emotional state or treatment burden.

The total number of days of admission was recorded for each patient.

### 2.4. Economic Analysis

Costs were compared between the two groups for LoS, cost of pumps (i.e., SIP and spring-infusor), and cost of preparation and administration of antibiotics. See Appendix A for more details of economic analysis.

### 2.5. Statistical Analysis

The Welch two sample *t*-test was used to assess between-group differences in age and FEV_1_ levels at admission and differences in step counts, sleep time, count of sit–stand motions, and standing and sitting times. The Wilcoxon test (deemed appropriate based on small sample size and evidence of skewed distribution) was used to assess the between-group difference in LoS. Bivariate relationships (for continuous data) were assessed using Spearman's rho (correlation coefficient). The beta coefficients from multivariable linear regression models with additive effects were used to estimate the average between-group difference in step count controlled for age and difference in LoS controlled for age and admission FEV_1_pp. Data were recorded in REDCap [13] and analysed in R Version 4·02 (R Project for Statistical Computing, Vienna, Austria) within the RStudio integrated development environment (Rstudio Team, Boston, MA).

## 3. Results

Thirty eligible patients were admitted during the study period. Of those, 27 patients were approached, and 22 consented. Two participants subsequently withdrew due to research study and dressing fatigue. Data for four participants was excluded (*n* = 1, device data error; *n* = 3, participants transferred to hospital in the home). The data error was due to an unknown malfunction when the data from the ActivPAL4 was downloaded. Full (final 5 days) data were captured for 16 participants: 10 females, mean (SD) age 14.5 (2.1) years, 80% (18%) initial FEV_1_. Of these 16 participants herein reported on, the 9 participants who opted to use the spring-infusor, tended to be older (15.7 (2.4) versus 12.9 (2.9) years age, *p* = 0.07), have higher FEV_1_ levels on admission (86% (11%) versus 72% (20%), *p* = 0.12), and shorter LoS (12.4 (1.7) versus 16.1 (4.3) days, *p* = 0.04) (Table 1).

**Figure 1:**
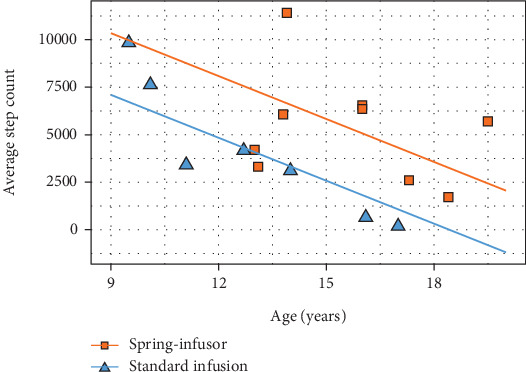
Average step count for each group by age.

Average step count was negatively associated with age (Spearman's rho = 0.50) and was higher, though not statistically significant, in participants using spring-infusors (mean 5324 (SD 2873) steps) versus SIP (4806 (3116) steps) (Table 1). Differential variation in step count was particularly evident when the device was considered jointly with age (Figure 1). We observed a mean (95% CI) increase in spring-infusor group compared to SIP of 3246 (54–6438) steps (*p* = 0.05) with an average decrease of 753 (188–1317) steps per year in age (*p* = 0.01). Step count did not correlate with duration of stay nor FEV_1_ at admission (Spearman's rho < 0.1); sleep time, sit–stand count, standing time, and sedentary times were also similar between groups. LoS was shorter on average in the spring-infusor group, (age-adjusted, *p* value 0.03; mean (SD) LoS: 16.1 (4.3) vs. 12.4 (1.7)).

**Figure 2:**
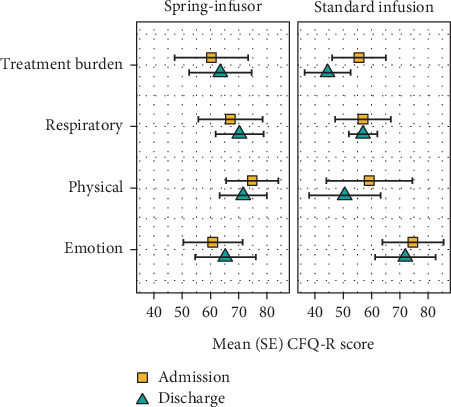
Admission and Discharge scores for CFQ-R by infusion delivery type. Figure legend: SE = standard error, CFQ-R = cystic fibrosis questionnaire-revised.

Quality of life data at admission and discharge were available for seven children on spring-infusors (mean (SD) age 15.6 (2.3) years) and six children using SIPs (13.2 (3.1) years). Small improvements in scores, on average, were seen in the spring-infusor group for the treatment burden and emotion domains, in contrast to declining scores in the corresponding domains observed for those using the SIPs (Table 1; Figure 2). Physical function was rated poorly across both groups and did not improve by discharge.

Data from the patient's diary were used to determine sleep time and cross-check with the ActivPAL4 sleep time. There was no significant difference in patient reports and ActivPAL4. No further data were used from the diary, as no patients removed the device.

### 3.1. Economic Analysis

After adjustment for age and initial FEV, children in the SIP group stayed an average of 2.4 days longer than those in the spring-infusor group. There was no difference in mean cost per day in hospital between groups with cost per day (across both groups) ranging between AUS$4400 and AUS $5900; thus, using a conservative estimate of AUS $5000 cost per day of admission, the estimated saving with spring-infusor treatment (from the average reduced LoS; 2.4 days) is, therefore, AUS$12000 as a result of reduced LoS.

The breakdown of costs for the two methods of infusion is presented in Appendix A Table A1 and Table A2, and details of costings are provided in the supplement. There were 110 exacerbations requiring admission per year. Using a conservative estimate of half being eligible for spring-infusors and based on data from this study, total annual savings would exceed AUS$0.5 million (calculation based on a reduction of inpatient stay by 2.4 days, a cost per day of AUS$5000 and 55 eligible subjects—total saving $660,000.)

## 4. Discussion

In this observational cohort study, children and young people hospitalised for a respiratory exacerbation of CF who were administered IVAB through a spring-infusor device were more active and had a shorter hospital stay than those administered IVAB through a SIP.

Given the spring-infusor group of participants stepped further than those using a SIP, the use of such a device may indeed promote physical activity during hospital admissions. Incorporating physical activity into treatment programs seems critical, given the known effectiveness of exercise training programs in children with CF [7] and management guideline recommendations to promote habitual physical activity and exercise into the daily treatment programme [16], including during hospital admissions [17]. Overall, we found that children admitted with an exacerbation of CF stepped on average 4800 steps a day, which is well below Australian recommendations [18]. To our knowledge, this is the first study to quantify activity levels in children with CF admitted to the hospital.

We previously found that patients preferred spring-infusors over SIPs for IVAB [4]. Patients believed that the small and simple device allowed for greater mobility, a reduction in unnecessary alarms, and improved patient's sense of wellbeing through increased autonomy during their admission and reduced focus on “feeling sicker,” through connection to a large SIP rather than a small, discrete portable device [4]. The current study provides further support for these previous qualitative study findings by demonstrating evidence of higher step counts and stable emotional wellbeing scores in those patients using a spring-infusor compared to those using a SIP.

While it is important to be cautious about drawing conclusions from this observational study, there were interesting trends in the two groups. There was evidence of stability or improvement in the spring-infusor group and regression (of treatment burden, emotional wellbeing, and physical wellbeing) in the SIP group. The SIP group had higher emotional wellbeing scores on average than the spring-infusor group at the start of their hospital admission, but the SIP group's emotional wellbeing scores worsened by the end of admission. The SIP group also had decreasing scores for the treatment burden by discharge. However, both groups' physical wellbeing scores did not improve over the admission, which may highlight patient's feeling deconditioned due to prolonged hospitalisation periods. The lack of improved physical wellbeing may reflect less time doing physical activity than usual for patients admitted with CF exacerbations [5].

It is important to note that all patients who used spring-infusors in the study chose this mode. Therefore, we could surmise that one reason for stable emotional outcomes and reduced treatment burden reported in their CFQ-R scores is partly due to their device choice, reflecting a benefit of patient-centred care, where patients have greater autonomy and are actively involved in their medical care, resulting in improved health outcomes [9, 19]. Importantly, patients' awareness of and experience with the spring-infusor device was dependant on the device's availability and the attitude of the nursing staff towards using the device. In this context, favourable nursing attitudes towards and availability of the device would likely improve patient experience and satisfaction. As previously reported, the spring-infusor is preferred by people with CF and allows for greater mobility with less attention to the patient feeling “sick.” Indeed, one participant suggested that “…if you're…attached to something for too long, you just feel…more sick…you're kind of stuck. A type of sick reality” [4]. For those patients who used spring-infusors, the provision of this device alone may have improved their mood, general wellbeing, and likelihood of having improved health outcomes and therefore lower LoS.

The importance of any potential benefits to patient-centred care cannot be understated, particularly in view of the growing evidence of improved health outcomes [20]. The idea that a relatively simple and portable device may provide such improvements in health outcomes is encouraging. The spring-infusor may also provide an economic health cost saving. A projection of more than half a million dollars a year is sizeable, based on just half of those children using the device.

This study has several limitations. As a small, observational study, patients were not randomised. Hence, we could not control for confounding factors such as disease severity (as measured by lung function), comorbidities, and age. COVID-19 may have impacted patients' ability to move, as the hospital management-imposed restrictions on hospital mobility. Further, children with CF are required to avoid contact with other children with CF to reduce the likelihood of cross-infection [21]. However, the study was conducted during the pandemic, and the restrictions for both COVID-19 and CF patients, more broadly, were uniform for both groups.

In conclusion, our study demonstrated that patients admitted with an exacerbation of CF were more active if they received their IVs via portable spring-infusor versus a SIP, and this was associated with a reduced length of hospital stay. Further research would provide stronger data to substantiate these findings.

## Figures and Tables

**Table 1:** Summaries of participant clinical characteristics, activity measures, and change in cystic fibrosis quality of life measures.

	**Overall** **N** = 16	**IV pump type**	
		**Spring-infusor** **N** = 9	**Standard infusion** **N** = 7
Demographic and clinical characteristics			
Age (years)	14.47 (2.90)	15.67 (2.38)	12.93 (2.91)
Initial ^1^FEV1 (%)	80 (17)	86 (11)	72 (20)
Change from admission in ^1^FEV1 (%)	8 (9)	4 (8)	12 (8)
Length of stay (days)	14.1 (3.5)	12.4 (1.7)	16.1 (4.3)
^2^Activity measures			
Average step count	4806 (3,116)	5324 (2873)	4139 (3512)
Sleep time (hours)_ave	9.06 (1.29)	9.00 (1.71)	9.14 (0.50)
Number of sit_stand_ave	37 (18)	37 (10)	37 (25)
Standing (hours)	1.96 (0.99)	2.22 (0.74)	1.61 (1.23)
Change in ^3^CFQ-R scores (discharge–admission)	*N* = 13	*N* = 7	*N* = 6
Treatment burden	−3 (23)	3 (25)	−11 (20)
Respiratory	2 (16)	3 (12)	0 (21)
Physical	−6 (17)	−3 (7)	−9 (26)
Emotion	1 (11)	4 (5)	−3 (16)

*Note:* Values are mean (standard deviation).

^1^FEV1: forced expiratory volume as a percent of predicted volume.

^2^Obtained from an ActivPAL4 activity monitor.

^3^CFQ-R: cystic fibrosis quality of life questionnaire.

**Table 2:** Cost of pumps including preparation and administration of antibiotics.

	**SIP**	**Springfusor**
Cost of pumps	AUS$164—2 years AUS$132 pa	AUS$110—2years [9] AUS$55 pa AUS$130—2 years AUS$65 pa

Antibiotics	Antibiotic regimens are determined clinically and not by the type of pump used	

Tubing	AUS$103 for box of 100—using 2 per day—approx. AUS$2 per day	AUS$430 for 50 or AUS$8.60 each. Replaced every 96 h -approx. AUS$2 per day

Syringes	Required by both delivery methods—no difference assumed	

Nursing time	15 min × 2 nurses	12–13 min × 2 nurses

*Note*: $ = AUD.

**Table 3:** Nursing time related to the use of different infusion devices.

	**SIP**	**Spring-infusor**
Preparing and administering antibiotics (dose prepared by Pharmacy)	Loading SIP, changing extension tubing, and priming the line with each dose (single use) also takes only 1–2 min	Loading spring-infusor and priming the line (1–2 min) Control tubing lasts 36 h
	Programming SIP (2 × nurses × 2–3 min)	No programming required
	Total time 15 min × 2 nurses	Total time 12–13 min × 2 nurses

Tobramycin preparation	2×nurses × 15 min	

Work created by alarms	SIP—give 5 min and 2 min warning alarms—loud and disruptive often means nursing staff has to wait in the room for 5 min prior to completion to silence the alarms and demand immediate response.	Spring-infusor has no alarms. This lack of alarm, however, can delay the necessary postdose flushing of the line

## Data Availability

Data is available upon request to the corresponding author.

## Appendix A: Economic Analysis

All costs were related to the 2020/21 financial year (Australian financial year runs from July 1, 2020, to June 30, 2021).

The administration timing for antibiotics was calculated by measuring the time taken for an experienced (≥ 5 years) paediatric nurse to complete its administration. We calculated the time by measuring three nurses performing both methods of infusion and calculated the mean time between the three nurses for each infusion.

In summary, both spring-infusors and SIPs can be used multiple times and replaced every 2 years with similar costs between the two groups. We assumed the cost of antibiotics did not differ between groups as both infusion methods used the same syringe, prepared in the same way by the pharmacist. Tubing costs and syringe costs showed minimal difference between infusion methods. Time taken by nurses was less for spring-infusors compared with SIP. However, more data would be needed to ascertain user performance variability and the potential for outliers to establish accurate data for nursing time.

## Nomenclature

CF: cystic fibrosis
HiTH: hospital in the home
IVAB: intravenous antibiotics
PCH: Perth Children's Hospital
SIP: standard infusion pump
